# Effects of Different Freezing Treatments during the Winemaking of a Varietal White Wine with Regard to Its Phenolic Components

**DOI:** 10.3390/foods12101963

**Published:** 2023-05-11

**Authors:** Juan Vilar-Bustillo, Ana Ruiz-Rodríguez, Ceferino A. Carrera, Zulema Piñeiro, Miguel Palma

**Affiliations:** 1 Department of Analytical Chemistry, Center of Agri-Food and Wine Research (IVAGRO), Faculty of Science, University of Cadiz, 11510 Puerto Real, Spain; juan.vilarbustillo@alum.uca.es (J.V.-B.); miguel.palma@uca.es (M.P.); 2 IFAPA Rancho de la Merced, Carretera de Trebujena, Km. 2.2, Apdo. 589, 11471 Jerez de la Frontera, Spain

**Keywords:** phenolic compounds, cryoextraction, pre-fermentative maceration, muscat grapes

## Abstract

In white wine production, the technique consisting of freezing whole or crushed grapes usually increases the levels of aroma-related compounds in the final wine products. However, this technique may affect phenolic compounds, among other chemical compounds. Phenolic compounds are crucial to white wines because of their susceptibility to oxidation and their role with regard to color stability. In this study, white wines made from Muscat of Alexandria grapes were subjected to two different freezing techniques: whole-bunch freezing and crushed-grape freezing. In addition, a pre-fermentative maceration was applied to each experiment in order to determine if the effects of freezing were comparable to those of maceration. The phenolic compounds studied were gallic acid, protocatechuic acid, caffeic acid, *trans*-coutaric acid, and epicatechin, which are the key compounds from the point of view of wine stability. The freezing of crushed grapes enhanced the extraction of phenolic compounds in comparison to the freezing of whole bunches of grapes without pre-fermentative maceration. On the other hand, the effect of pre-fermentative maceration was comparable to that resulting from freezing crushed grapes. This step made the must from whole frozen grapes having even larger levels of phenolic compounds. Without pre-fermentative maceration, freezing whole bunches of grapes only allowed a moderate extraction of phenolic compounds and produced wines with lower individual phenolic contents than those obtained through traditional winemaking procedures.

## 1. Introduction

Most of the chemical compounds responsible for the varietal aroma in white wines are contained in the skins of the grapes used. Pre-fermentative maceration, prior to alcoholic fermentation, is one of the methods used to promote their extraction as maceration allows the solid parts of grapes to be in contact with the must. However, maceration can also increase the levels of phenolic compound content, and this particular fact must be closely controlled when making white wines as it may affect the sensory profile and stability of the final wine products [1]. Phenolic compounds are responsible, among other chemical products, for the occurrence of oxidation processes that modify some of the organoleptic properties, such as the aroma and the color of wines [2]. For this reason, macerations performed on white wine are carried out at low temperatures to increase the concentration of aromatic compounds in the grape juice and, at the same time, limit the extraction of phenolic compounds [3]. For instance, it has been demonstrated that a 6 h pre-fermentative maceration at 10 °C enhanced the recovery of aroma-related compounds, i.e., alcohols, terpenes, ethyl esters, and acetate esters. Although it also increased the concentration of phenolic compounds, the increment was not enough to significantly alter the bitterness or astringency of the final wine in comparison to wines that had not been subjected to pre-fermentative maceration [4]. Nevertheless, in another similar study, with the white grape variety, Vugava, maceration at the same temperature and using two different times (10 and 20 h) caused no effect, neither on phenolic compounds nor on wine aromas [5]. Even red wines are also often produced using pre-fermentative maceration. For instance, Cabernet Sauvignon grapes were subjected to pre-fermentative maceration for 3 to 7 days at 5 to 8 °C. The result was a change in the composition of volatile compounds of the final wines as a consequence of their lower C6 alcohol and fatty acid concentrations [6]. A similar study on the same grape variety and using low-temperature maceration resulted in more aromatic wines with a lower content of phenolic compounds [7]. Therefore, it has been demonstrated that the effects of maceration can vary depending on the grape variety and may affect not only the levels of phenolic compounds but also the formation of other compounds, such as amino acids, vitamins or fatty acids, that come from the grapes used, as well as the metabolism of yeasts during the fermentation process. All of these can have a definite effect on the organoleptic characteristics of wines.

Another method that has been used to improve the extraction of aromas consists of incorporating ultrasounds into the maceration process. Thus, the white grape variety, Muscat of Alexandria, was subjected to 4 h pre-fermentative maceration at 4 °C and ultrasound treatments for 40 to 80 min. The wines obtained through these experiments were more aromatic but did not have a greater concentration of phenolic compounds [8]. It means that appropriate conditions can favor the selective extraction of aromatic compounds, without increasing the levels of phenolic compounds.

Freezing is another alternative method that promotes the recovery of compounds from grape skins; its difference from pre-fermentative maceration consists of lowering the temperature to freeze the grape bunches or the paste formed after crushing. As grape tissues freeze and ice crystals are formed, these break down cell walls, which allows additional extraction from the grapes. Later, any of the previously cited techniques to enhance the recovery of compounds from grape skins can also be applied. Additional compounds extracted from the freezing step confer a greater varietal character to the final wine [9]. For example, liquid carbon dioxide was used with the Italian white grape variety, Greco, in order to lower the maceration temperature to 2–4 °C for 24 h. As a result, the final wines acquired a more pronounced varietal character. These wines also presented a greater volatile fraction and higher concentration of phenolic compounds, which increased their aromatic complexity and enhanced protection against oxidation [10]. The same procedure was applied to other Italian varieties, namely Falanghina and Bombino bianco, with similar results with regard to varietal aromatic composition and phenolic compounds, which resulted in very singular wines with a superior antioxidant capacity, volume, and presence in the mouth [11]. A similar trial was conducted on the white grape variety, Sauvignon blanc, with a 24 h pre-fermentative maceration using liquid carbon dioxide. This treatment achieved a higher level of phenolic compounds in the wine and enhanced its antioxidant activity, thereby preserving its varietal profile. On the other hand, the freezing treatment did not induce the formation of quinones, which are compounds responsible for the brown color of white wines [12].

A study was conducted on the effect of freezing whole Muscat of Alexandria grapes at different speeds, i.e., either using liquid nitrogen or a freezing chamber. In both cases, and particularly when using liquid nitrogen, more aromas were extracted, specifically terpenes and fatty acids, while no noticeable adverse effects on other compounds of interest were observed [13]. Additionally, the effect of freezing Palomino Fino grapes at −18 °C versus regular pre-fermentative maceration was investigated. The freezing technique was the one that produced wines with the most aromatic profile, and they also exhibited a more intense taste [14].

The present work investigates the effects of freezing on the winemaking of a specific dry white wine. Two different freezing techniques were applied to grapes of the Muscat of Alexandria variety as follows: whole-bunch freezing and crushed-grape freezing. Furthermore, two of the frozen musts were subjected to pre-fermentative maceration, while the other two frozen musts did not undergo pre-fermentative maceration. This resulted in the necessary reference assays that allowed the determination of whether the effect of freezing was comparable to that of pre-fermentative maceration. The data with respect to the different effects on the wines’ volatile compounds have been recently published [15]. It was found that the wines produced after whole grapes were frozen showed higher aromatic concentrations compared to the wines obtained by either freezing crushed grapes or the wines obtained with traditional winemaking techniques. Additionally, the compounds affected by freezing either whole bunches or crushed grapes were terpenes, acids, and esters. Lower differences were found for wines produced by applying pre-fermentative maceration after the freezing process. Therefore, because the freezing procedures produced very interesting wines, the present work addresses the different effects observed with respect to the phenolic content of the wines produced so that the most appropriate processing conditions can be determined.

## 2. Materials and Methods

### 2.1. Grape Variety and Winemaking Procedures

Muscat of Alexandria grapes were cultivated at Chiclana de la Frontera, Cadiz (lat. 36°25′48.5″ N, lon. 6°06′30.1″ W). The grapes were harvested when they reached 23.2° Brix sugar contents. The total acidity was 3.6 g L^−1^ of tartaric acid. The stems had not lignified yet. The grapes were divided into three batches, each of which was subjected to different winemaking treatments as follows:

- Reference batch treatment (R): the grape bunches were destemmed and traditionally milled, and no freezing process was used;
- Crushed-grape freezing (M): the bunches were destemmed, the grapes were crushed, and the must was frozen in a chamber at −18 °C;
- Whole-bunch freezing (B): the bunches of grapes, without any further processing, were frozen in a chamber at −18 °C.

For the M and B batches, the grapes remained in the freezing chamber for 14 days.

These batches were then divided into two sets: for the first set, a 4 h pre-fermentative maceration at 10 °C was conducted, whilst the second set was processed without maceration. Later, the same winemaking processes were applied to the two sets. All trials were carried out in triplicate. Table 1 shows the different winemaking procedures and the codes used for their identification.

After pressing the grapes and stabilizing their temperature, the 18 trials (6 winemaking conditions in triplicate) were treated in the same way, i.e., the liquid was separated from the solids. Steel containers (50 L, diameter: 30 cm, and height: 70 cm) were filled up with approximately 45 L for the fermentation process. Tartaric acid was used to fix the pH of the must at 3.3, as recommended for the specific yeast that was going to be used for the alcoholic fermentation of the wines. Sulphur dioxide (SO_2_) was added in the form of potassium metabisulphite until a concentration of 40 mg L^−1^ was reached.

Active dry yeast, specifically *Saccharomyces cerevisiae* var. Bayanus VINIFERM Revelacion (Agrovin, Alcazar de San Juan, Spain), was used for the alcoholic fermentation process, which was carried out in a cold chamber at 16 ± 1 °C for 10 days. The yeast was prepared according to the instructions provided by the supplier: a ratio of 100 mg of yeast per liter of the must was added to each fermentation tank. The fermentation process was considered completed when the content of reducing sugars dropped below 5 g L^−1^.

Finally, the wines were allowed to settle statically for eight days and filtered through cellulose plate filters with a 20 µm pore size. The wines were stored in 10 L bags in box deposits at 15 °C, with the addition of potassium metabisulphite until a sulfur dioxide concentration of 60 mg L^−1^ was obtained. All analyses were conducted in a period of 3 months after complete fermentation.

### 2.2. Characterization of the Wines

The analytical parameters of the musts and wines were monitored as follows: total acidity was determined using acid–base titration in a pHmatic 23 (Crison, Barcelona, Spain); sugar level was expressed as °Brix and determined using a DMA 4500 M densimeter (Anton paarGmBH, Graz, Austria); and ethanol content was determined following the regular method, i.e., after the wines were distilled, their density was determined using the above-mentioned densimeter [16].

### 2.3. Determining Total and Individual Phenolic Compounds

UV-Vis spectrophotometry was used to determine the different levels of total phenolic compounds using a Cary 60 UV-Vis (Agilent, Santa Clara, CA, USA) according to a method previously used [7]. Each determination was carried out in triplicate on each wine sample at 280 nm, after diluting it to a ratio of 1:10 with distilled water and using 10 mm light path quartz cuvettes. A calibration curve of known concentrations between 100 and 500 mg L^−1^ of gallic acid was used to quantify the phenolic compounds, and the results were expressed as mg L^−1^ of gallic acid equivalent.

Individual phenolic compounds were measured on a Waters Acquity H-Class UPLC coupled to a Photo Diode Array detector, using 3 µL samples as the injection volume. A BEH reversed-phase column (100 mm length; 2.1 mm I.D.; and 1.7 µm particle size) (Waters) at a temperature of 47 °C was used. The mobile phases were composed of acidified water (2% acetic acid) (solvent A) and 2% acetic acid in acetonitrile (solvent B), and a flow rate of 0.6 mL min^−1^ was used. The gradient used for the separation was as follows: 0 min 0% B, 1.0 min 0% B, 3.0 min 5% B, 4.0 min 10% B, 4.5 min 10% B, 5.0 min 20% B, 7.0 min 20% B, 8.0 min 30% B, 10.0 min 30% B, and 11.0 min 100% B [17].

Prior to the UPLC analysis, the samples were extracted using solid-phase extraction (SPE) [18,19]. Strata X 200 mg cartridges (Phenomenex, Torrance, CA, USA) were used. The cartridges were preconditioned with 5 mL of methanol followed by 5 mL of water. Then, the samples were loaded (25 mL). They were washed down using 10 mL of water and, finally, the elution was performed using 2 mL of methanol.

The concentrations of the different individual phenolic compounds were determined using the calibration curve of each phenolic compound (Table 2), with known concentrations between 0.2 and 17 mg L^−1^. Gallic acid, protocatechuic acid, *p*-coumaric acid, caffeic acid, and epicatechin were purchased from Sigma-Aldrich (St. Louis, MO, USA).

Caftaric acid and *t*-coutaric acid were quantified using the calibration curves of caffeic acid and *p*-coumaric acid, respectively, and the due molar ratios between the acids and the tartaric ester chemical forms.

### 2.4. Susceptibility to Oxidation

In order to evaluate the evolution and susceptibility of the wines to oxidation, 30 mL of the final wines from each assay were left in direct contact with air at room temperature for 14 days. Absorbance was measured at 420 nm wavelength every 24 h.

NestCell Culture Flask Canted Neck Polystyrene (Nest, Wuxi, China) cell culture flasks with treated surfaces and perforated stoppers were used to allow air to enter the flasks so that oxygen could be gradually in contact with the wines while contamination by microorganisms was prevented. This study was carried out in duplicate.

### 2.5. Statistical Analyses

Statistica (TIBCO Software, Palo Alto, CA, USA) was used to perform cluster analyses (CA) and to generate the resulting dendrograms. Analysis of variance (ANOVA) was conducted using the RStudio (RStudio, Boston, MA, USA) software to perform the pairwise tests.

## 3. Results

### 3.1. Effects of Freezing on the Wines That Did Not Undergo Pre-Fermentative Maceration

#### 3.1.1. General Characterization of the Musts and Wines That Did Not Undergo Pre-Fermentative Maceration

Table 3 includes the results corresponding to the characterization of the initial must and the final wines, along with the results from the Student’s *t*-test. In all cases, the wines produced were compared against the reference wine (the one that had not been frozen, i.e., R0).

Total phenolic compound levels were between 198.3 and 293.4 mg L^−1^. These values are comparable to those reported by other works published on other white grape varieties [5,12]. In our study, the must that had been frozen after crushing (M0) presented some significant differences with respect to the reference must, which had not been frozen (R0). M0 exhibited 36% higher total phenolic concentration compared to that of R0 (293.4 mg L^−1^ vs. 215.2 mg L^−1^). In contrast, the must from the whole-bunch freezing trial (B0) presented similar concentrations to those of the reference must (R0) (215.2 mg L^−1^ vs. 198.3 mg L^−1^).

On the other hand, the final wine from the assay using frozen crushed grapes (M0) was once again the sample with the highest concentration of phenolic compounds, even if the increases were lesser (12% higher than R0: 245 mg L^−1^ vs. 219 mg L^−1^) than those observed in the must, i.e., the wine obtained from the frozen whole grapes (B0) had the lowest concentration of total phenolic compounds (B0: 204.8 mg L^−1^).

This fact is particularly interesting as it implies that the increment in phenolic compounds only occurred in the case where crushed grapes had been frozen (M0), while no increase was recorded in the case of whole-grape freezing (B0). It should be assumed that the lower value could not be explained by a lower extraction but by the co-extraction of other compounds that favor the precipitation or degradation of phenolic compounds. A similar behavior had been reported, for example, in research studies on the Mencía red grape variety, where cold macerations were carried out at 5 °C for 2 and 6 days. This resulted in increased extractions of phenolic compounds, while this effect disappeared after aging in bottle [20,21]. In the same line, the effect of freezing Malbec grapes was studied, and it could be observed that the wines exhibited greater color intensities due to their higher contents of phenolic compounds. Nevertheless, these higher contents disappeared after aging in bottle [22].

#### 3.1.2. Characterization of Individual Phenolic Compounds in Wines without Pre-Fermentative Maceration

A set of six specific phenolic compounds were selected to evaluate the effects of the processing techniques. These compounds were selected based on their relationships with the oxidation processes in white wines and also in order to represent different phenolic families (benzoic acids, cinnamic acids, and flavanols) that are related to specific parts of grapes, i.e., grape pulp, skin, and seeds [23,24].

The results corresponding to the quantification of individual phenolic compounds in the wines that had not been subjected to pre-fermentative maceration are shown in Table 4. In general, these results are similar to those obtained for total phenolic compounds, although certain relevant particularities can be observed.

First, it should be noted that each phenolic compound behaves differently depending on the set up of the experiments. For example, protocatechuic acid reached a 3-fold higher concentration in the wine made with the freezing of crushed grapes (M0) relative to the reference wine (R0). However, the wine from the B0 assay only reached 1.5 times the amount of protocatechuic acid in R0. Therefore, the result is considerably less noticeable when freezing whole berries. However, the concentration of gallic acid in M0 was 1.5 higher than it was in R0, while it was twice as high in B0 (2.1 higher). Similar behaviors were observed in the study by Olejar et al., where the concentrations of individual phenolic compounds presented significant differences depending on the set up of the experiments and the phenolic compound involved [12].

On the other hand, it should be noted that extremely high levels of cinnamic compounds were found in M0. For example, caffeic acid was about five times higher in M0 with respect to the reference wine R0 (M0: 9.25 mg L^−1^ vs. R0: 2.00 mg L^−1^); *trans*-coutaric acid was four times higher in M0 with respect to R0 (M0: 14.25 mg L^−1^ vs. R0: 3.86 mg L^−1^); and caffeic acid had a concentration of 9.85 mg L^−1^ in M0, whereas R0 presented values below the quantification limit. This is explained by the easier extraction of phenolic compounds from the skin of frozen crushed grapes (M0) [25]. In contrast, the wines from frozen whole grapes (B0) presented really low concentrations. In certain cases, such as the B0 wines, both caffeic acid and *trans*-coutaric acid were present in lower concentrations that those normally found in the wines produced using regular winemaking process (R0). Thus, caffeic acid was found at 0.79 vs. 2.00 mg L^−1^ and *trans*-coutaric acid was found at 0.38 vs. 3.86 mg L^−1^ in B0 and R0, respectively.

It should be taken into account that the concentrations of individual phenolic compounds may vary according to several factors: the part of the grape in which they are found, the procedure applied, the evolution over time, and the types of phenolic compounds [26,27].

For example, the increment in epicatechin due to freezing was significant in M0, with a difference of 36% with respect to R0 (0.92 mg L^−1^ vs. 0.59 mg L^−1^). In the case of B0, non-significant difference was registered. Since epicatechin was found at higher concentrations in the seeds, the freezing treatment had a limited effect on the extraction of seed components when whole berries were frozen.

Caftaric acid, on the other hand, was detected in R0 and B0 at levels below the limit of quantification, while the assay that included the freezing treatment of grape pulp and skins allowed the quantification of this acid. This compound is found at greater concentrations in grape skin, although it can also be found in the pulp. The results suggest that the degradation suffered by grape skin during freezing facilitates its extraction into the must during the winemaking process. The differences that were found between the M0 wine (9.85 mg L^−1^) and the other wines (R0 and B0: traces below the limit of quantification) seemed to indicate that its content level in the berry skin was high. Either because of the lesser degradation of grape tissues from the freezing treatment or because of the processing conditions, which favored the enzymatic oxidation of this compound, using frozen bunches produced limited levels of caffeic acid in the final wines from the same grapes.

### 3.2. Effects of Freezing on the Wines Produced through Pre-Fermentative Maceration

#### 3.2.1. Evolution of Phenolic Compounds during the Pre-Fermentative Maceration of the Wines

**Figure 1:**
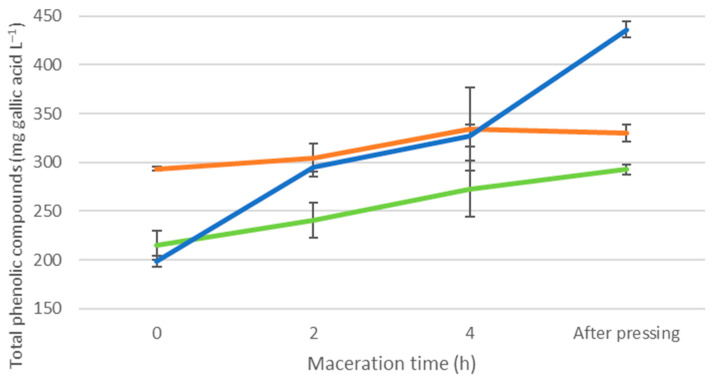
Evolution of total phenolic compounds (mg L^−1^ of gallic acid) during the 4 h of pre-fermentative maceration.

Figure 1 shows the evolution of the concentration of the phenolic compounds during the pre-fermentative maceration of the musts. The results of the three experiments, at 0 h, reflect the effects of freezing as previously displayed in Table 3. Along with maceration, their concentrations increased, even though the increase occurred differently according to the assay procedures.

The reference trial RC, which had not undergone the freezing stage, experienced an increment in the concentration of phenolic compounds by 12% after 2 h of pre-fermentative maceration, 27% after 4 h, and 36% at the end point, just after pressing.

The BC trial, which grapes had been frozen, increased its concentration of phenolic compounds by 48% after 2 h of pre-fermentative maceration, which was 4 times higher than the increment experienced in the RC trial (12%). This increment was large enough to reach, from this point on, significant differences with the RC must (294 mg L^−1^ vs. 240 mg L^−1^). After 4 h of maceration, the increase was 65% and, after pressing, it reached 120%, which exceeded that of RC by 49% (436 mg L^−1^ vs. 292 mg L^−1^) after the pressing process.

The MC trial, where freezing was applied to crushed grapes, had a significantly higher initial content of phenolic compounds than any other musts, whether RC or BC. However, during pre-fermentative maceration, it registered smaller increments. Thus, after 2 h, it had gone up by just 4%, and after 4 h, it reached 14%, with no additional increase being found after the pressing process. Thus, the overall increase was 14%, while in the RC must, the increment was more than double, with both experiments having similar concentrations at the end of the pre-fermentative maceration process (330 mg L^−1^ of MC vs. 293 mg L^−1^ of RC).

When comparing the two musts from the freezing assays, i.e., MC vs. BC, after 2 h of maceration, the BC must reached a similar polyphenolic content as that of the MC must (299 mg L^−1^ vs. 304 mg L^−1^). Right after pressing, the BC must significantly outperformed the MC must by 32% (436 mg L^−1^ vs. 330 mg L^−1^) and reached significantly higher levels of polyphenolic contents with respect to the other three experiments.

It can be said that the pre-fermentative maceration process substantially modifies the content levels of phenolic compounds and increases them in the three cases tested, even though the variations are substantially different. These differences mean that the effects resulting from freezing crushed grapes disappear, and in contrast, they appear in the musts obtained through the freezing of whole bunches of grapes. This can be explained by a greater capacity to extract phenolic compounds from the skins of frozen whole grapes, whereas this does not occur when crushed grapes get frozen. It means that depending on how the freezing technique is applied, maceration will have a different outcome, which will only be significant when whole grapes are frozen.

In light of these results, it can also be concluded that the lower total phenolic compound contents in frozen musts without pre-fermentative maceration are simply due to lower extraction, rather than to any type of reaction or degradation. Hence, by applying pre-fermentative maceration, these compounds can be extracted, and their concentration will increase.

#### 3.2.2. Characterization of the Musts and Wines after Pre-Fermentative Maceration

Table 5 shows the content levels of phenolic compounds in the musts and wines after their pre-fermentative maceration.

The results for the musts are the same as those shown in Figure 1, with significant differences only in the case of the BC must. Once the winemaking process had been completed, the behavior of the initial musts in the trials changed with respect to the final wines. The BC wine presented no significant differences with respect to the RC wine in terms of total phenolic compounds (223.5 mg L^−1^ vs. 213.9 mg L^−1^). On the other hand, the MC wine showed significantly different levels with respect to the RC wine.

The difference in the behavior of phenolic compounds in the must before fermentation and in the finished wine is explained by the oxidation and precipitation reactions of the phenolic compounds that take place during alcoholic fermentation and wine stabilization. Thus, these reactions lead to the elimination of some of the phenolic compounds. This effect had already been reported in other studies, such as the study by Nenadis and Paraskevopoulou [28] on the evolution of oxidation in seven commercial white wines. It was concluded that despite having the same origin, the oxidation behavior in the wines differed due to the subsequent conditions that the wines had been exposed to. In our case, the content of total phenolic compounds of the BC wine was different from that of the MC wine, and this could explain why during the fermentation and stabilization of the BC wine, there was a greater drop in phenolic compounds.

#### 3.2.3. Characterization of Individual Phenolic Compounds in the Wines Subjected to Pre-Fermentative Maceration

Table 6 shows the quantification results of individual phenolic compounds in the wines produced using pre-fermentative maceration.

The wines in which crushed grapes were frozen (MC) are the ones that present the highest concentrations of individual phenolic compounds compared to those obtained from freezing whole grapes (BC) or those produced through traditional winemaking procedures (RC). Specifically, extremely high levels of cinnamic derivatives were found, i.e., caffeic acid level was six times higher than in the RC wine (MC: 9.13 mg L^−1^ vs. RC: 1.44 mg L^−1^), caftaric acid level was at least eight times higher (M0: 8.81 mg L^−1^ vs. RC: traces) and *trans*-coutaric acid level was close to four times higher than in the RC wine (MC: 9.95 mg L^−1^ vs. RC: 2.67 mg L^−1^). This is explained by the easier extraction of phenolic compounds from the skins of grapes that had been crushed and frozen (MC). In contrast, in the wine obtained from whole frozen grapes (BC), despite its substantially high extraction yield during its pre-fermentative maceration (Figure 1), where total phenolic compounds increased by 93% in the must, the final wine had really low phenolic compound contents. These results coincide with the trend for total phenolic compound contents in the wines (Table 4). This is attributed to the oxidation and co-precipitation reactions that eliminate phenolic compounds, particularly cinnamic acids. This effect had already been reported in studies where winemaking procedures that involved greater aeration and contact with oxygen caused the losses of cinnamic compounds [28,29].

In general, as shown in Table 6, significant differences in the levels of individual phenolic compounds are registered for the different experiments, as is the case for the wines that did not undergo pre-fermentative maceration. For example, with respect to protocatechuic acid, the MC wine is the only wine with a value higher than the LOQ (1.17 mg L^−1^). It should be noted that this is best reflected by *trans*-coutaric acid since its different content levels do not accord with whether pre-fermentative maceration was applied (RC, MC, and BC) (Table 5) or was not applied (R0, M0, and B0) (Table 3), given that the concentrations are different in the wines from all six trials. In fact, this behavior had already been described in other studies, such as the study by Olejar et al., where the concentration of specific individual phenolic compounds exhibited significant differences according to the experimental conditions and the particular phenolic compounds being measured [12].

However, with regard to the rest of the individual phenolic compounds, it was observed that pre-fermentative maceration of the wines minimized any of the differences caused by the freezing stage. Thus, when comparing the results in Table 4, it can be seen that the traditionally produced wine has doubled its content of gallic acid with respect to that of the wines that had not undergone pre-fermentative maceration (RC: 0.83 and R0: 0.45 mg L^−1)^. In contrast, regarding the same compound, the BC wine does not differ from the M0 wine (0.61 vs. 0.67 mg L^−1)^.

On the other hand, epicatechin did not show any differences in the RC wine, which had been made following traditional pre-fermentative maceration, when compared against the R0 wine, which had not undergone this process. However, the two freezing experiments did show differences with respect to those including pre-fermentative maceration. Specifically, the MC wine exhibited half of the concentration of epicatechin when compared to the M0 wine, while the BC wine presented a 10% higher content than the B0 wine. The explanation for this behavior is that epicatechin is mainly located in the seeds; thus, when grapes are crushed before the freezing phase (M0), greater amounts of epicatechin are transferred into the musts than in those cases where whole grapes are frozen (B0). However, pre-fermentative maceration, such as the case of the MC wine, did not increase epicatechin concentration levels beyond those already achieved through the freezing step.

Caftaric acid was not extracted in the traditional winemaking experiments, R0 and RC, but it could be quantified in the trials that involved a freezing stage. However, no increments were observed with pre-fermentative maceration; thus, it remained constant in the BC wine with no differences when compared to the B0 wine and decreased by 10% in the case of the MC wine with respect to the M0 wine.

**Figure 2:**
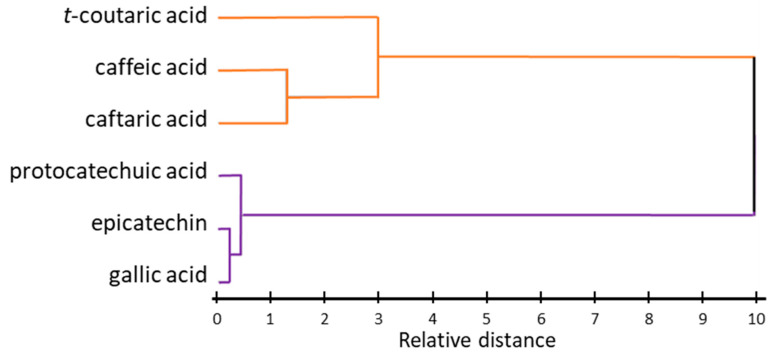
Dendrogram of individual phenolic compounds. Different colors are used for the two main groups of phenolic compounds that are formed after the cluster analysis.

A cluster analysis (CA) was performed to determine how similar the behavior of the different compounds was in relation to the winemaking techniques tested (Figure 2).

It can be seen from the dendrogram that there are two clearly differentiated large groups of individual phenolic compounds. On the one hand, we find the cinnamic compounds (caffeic acid, *trans*-coutaric acid, and caftaric acid) being grouped in the upper part of the dendrogram, since they are similarly distributed in the berries and are the most susceptible compounds to oxidation processes [30,31,32]. Most of these compounds are responsible for the browning of musts or wines as a result of the activity of polyphenol oxidase enzymes [31,32]. Gallic acid, epicatechin, and protocatechuic acid, on the other hand, even though they belong to different families of phenolic compounds, exhibit similar behavior and concentrations. This is explained by the fact that most of them are found in pulp and seeds. Therefore, the behavior of these individual phenolic compounds seems to be related to their chemical properties and, particularly, to their susceptibility to oxidation, as well as to their location in the different parts of the berries, i.e., skins or seeds.

### 3.3. Evaluation of Stability against Oxidation

A study on the susceptibility to oxidation of the finished wines was conducted in order to verify whether the freezing and pre-fermentative maceration processes that had been applied in the different experiments increased or decreased the oxidation of the wines. Susceptibility should be greater in those wines with higher phenolic contents, although their specific composition and the presence or absence of other compounds might also affect their susceptibility to oxidation.

**Figure 3:**
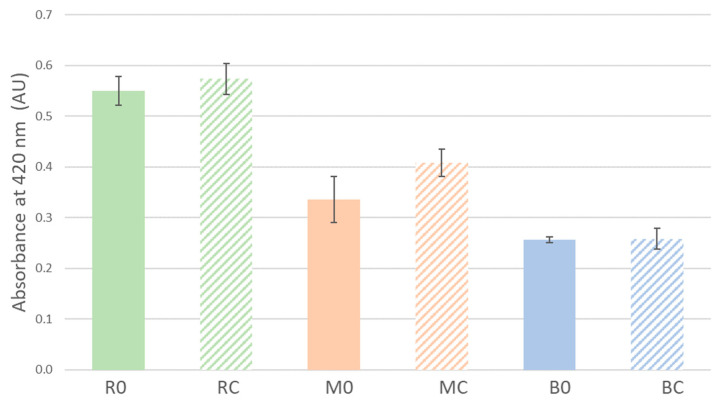
Absorbance of the final wines at 420 nm after 14 days. R: Regular winemaking process, with no freezing step. M: Winemaking process with the freezing of must (grape pulp and skins) after destemming and crushing. B: Winemaking process with the freezing of whole bunches of grapes. 0: Winemaking without pre-fermentative cold maceration. C: Winemaking with pre-fermentative cold maceration.

Figure 3 shows the behavior vs. the oxidation of the wines. The traditional winemaking experiment (R0) shows the highest absorbance after 14 days, followed by the one in which grapes had been crushed and frozen (M0), and finally the assay in which whole grapes had been frozen (B0), with significant differences. It shows that the freezing process increases protection against the oxidation of the wines by slowing down their evolution and in a particularly interesting manner in the case of wines made from frozen whole grapes (B0).

The wines that underwent pre-fermentative maceration during the winemaking processes (RC, MC, and BC) exhibit higher absorbance and more browning over time (Figure 3). On the other hand, it is worth recalling the results regarding the total phenolic compounds that were examined for the different winemaking processes. Thus, the crushed-grape freezing experiment (MC), having the largest levels of phenolic compounds, would be expected to present the highest susceptibility to oxidation and thus, more browning, followed by the wine produced through traditional winemaking (RC) and, finally, by the wine made with frozen whole grapes (BC). However, it turns out that this correlation between phenolic compound content and susceptibility to oxidation of the wines does not actually apply. It could, therefore, be concluded that, during the winemaking processes involved, the phenolic compounds that present a higher susceptibility to oxidation are eliminated in spite of being further extracted through the maceration process. This makes the final wines particularly stable, which implies that a larger level of phenolic compounds does not necessarily entail a higher susceptibility to browning. This stabilization effect is rather notable in the wines where grapes had been crushed and frozen (MC) and is even more pronounced in the case of whole-grape freezing (BC). The removal of the compounds that are most susceptible to oxidation could be the result of a more intense action by polyphenol oxidase enzymes. However, this has not been verified in this study. In any case, and most importantly, all the final wines which winemaking involved a freezing step exhibit a greater protection against oxidation than any of the reference wines.

## 4. Conclusions

First, it must be noted that the freezing period used in this work was long in order to detect the effects of this procedure in the musts and wines. Shorter periods should be evaluated by winemakers on specific grape varieties. Using this specific period, it was observed that the freezing treatments favored the extraction of phenolic compounds from the skin to the must. The freezing of crushed grapes was the process that most favored the extraction of phenolic compounds if pre-fermentative maceration was not applied. Freezing whole grapes produced wines with a lower concentration of total phenolic compounds than the reference wines, even though the BC wine was made from a must with a higher amount of total phenolic compounds than the reference musts. This difference was particularly marked with regard to cinnamic compounds.

On the other hand, the use of pre-fermentative maceration increased the concentration of phenolic compounds, but it had different effects depending on the pretreatment of the samples. In any case, pre-fermentative maceration reduced the differences resulting from the freezing treatments employed.

The wines obtained after freezing whole grapes and without applying pre-fermentative maceration were particularly interesting as they showed the lowest contents of phenolic compounds.

## Figures and Tables

**Table 1:** Details of the six winemaking procedures used.

Must Batch	Description
R0	Regular winemaking process. Destemming of the bunches and crushing of the grapes, followed by immediate pressing and racking
M0	Frozen must with crushed grapes, i.e., destemming and crushing of the grapes followed by the freezing of their pulp and skins. After thawing, the grapes were pressed and racked.
B0	Freezing of whole bunches of grapes. After thawing, the grapes were destemmed and crushed, followed by their immediate pressing and racking.
RC	Destemming and crushing of the grapes, followed by cold pre-fermentative maceration of the paste for 4 h at 10 °C. Pressing and racking were performed after the cold maceration.
MC	Destemming and crushing of the grapes, followed by freezing of the grapes’ pulp. After thawing, cold pre-fermentative maceration was performed for 4 h at 10 °C. Pressing and racking were performed after the cold maceration.
BC	Freezing whole bunches of grapes. After thawing, the grapes were destemmed and crushed and then cold macerated for 4 h at 10 °C. After their maceration, the grapes were pressed and racked.

**Table 2:** Calibration lines, linear regression coefficients, and detection limits of the individual phenolic compounds.

Compound Name	Regression Equation	Squared Regression Coefficient (*R*^2^)	LOD (mg L^−1^)	LOQ (mg L^−1^)
Gallic acid	y = 30,593x + 1463	0.9999	0.14	0.43
Protocatechuic acid	y = 34,822x + 9115	0.9992	0.10	0.34
*p*-Coumaric acid	y = 123,270x + 38,070	0.9994	0.11	0.35
Caffeic acid	y = 40,016x + 19,027	0.9985	0.21	0.71
Epicatechin	y = 660,659x + 283,654	0.9997	0.13	0.41

**Table 3:** Phenolic compounds found in the musts and wines (*n* = 3). An asterisk indicates a significant difference (*p* < 0.05) from the control wine, which had not been subjected to any freezing procedure (R0).

	Total Phenolic Compounds (mg L^−1^ of Gallic Acid)	
	Musts	Wines
R0	215.2 ± 7.5	219.5 ± 7.1
M0	293.4 ± 14.9 *	245.1 ± 3.1 *
B0	198.3 ± 6.1	204.8 ± 5.6

R: Regular winemaking process, with no freezing step. M: Winemaking process with the freezing of must (grape pulp and skins) after destemming and crushing. B: Winemaking process with the freezing of whole bunches of grapes. 0: Winemaking without pre-fermentative cold maceration.

**Table 4:** Concentration of individual phenolic compounds ^1^ in the final wines (mg L^−1^).

	R0	M0	B0
Gallic acid	0.45 ± 0.01 ^a^	0.67 ± 0.07 ^b^	0.93 ± 0.12 ^c^
Protocatechuic acid	0.41 ± 0.04 ^a^	1.24 ± 0.04 ^b^	0.61 ± 0.03 ^c^
Caftaric acid	Traces ^2^	9.85 ± 0.07 ^a^	Traces ^2^
*t*-Coutaric acid	3.86 ± 0.06 ^a^	14.25 ± 0.05 ^b^	0.38 ± 0.02 ^c^
Caffeic acid	2.00 ± 0.02 ^a^	9.25 ± 0.03 ^b^	0.79 ± 0.08 ^c^
Epicatechin	0.59 ± 0.10 ^a^	0.92 ± 0.14 ^b^	0.69 ± 0.02 ^a^

^1^ Different letters in the same row indicate significant differences at 95% confidence according to the ANOVA and pairwise test. ^2^ Content below the LOQ. R: Regular winemaking process, with no freezing step. M: Winemaking process with the freezing of must (grape pulp and skins) after destemming and crushing. B: Winemaking process with the freezing of whole bunches of grapes. 0: Winemaking without pre-fermentative cold maceration.

**Table 5:** Main parameters of the starting musts and the final wines (*n* = 3).

	Total Phenolic Compounds (mg L^−1^ of Gallic Acid)	
	Musts	Wines
RC	293.0 ± 6.4 ^a^	213.9 ± 7.4 ^a^
MC	330.6 ± 10.8 ^a^	245.4 ± 4.9 ^b^
BC	436.0 ± 12.1 ^b^	223.5 ± 5.8 ^a^

R: Regular winemaking process, with no freezing step. M: Winemaking process with the freezing of must (grape pulp and skins) after destemming and crushing. B: Winemaking process with the freezing of whole bunches of grapes. C: Winemaking with pre-fermentative cold maceration. In the same column, different letters mean significant differences (*p* < 0.05).

**Table 6:** Quantification of individual phenolic compounds in the wines produced with pre-fermentative maceration (mg gallic acid L^−1^) ^1^.

	RC	MC	BC
Gallic acid	0.83 ± 0.09 ^a^	0.61 ± 0.06 ^b^	0.94 ± 0.03 ^c^
Protocatechuic acid	Traces ^2^	1.17 ± 0.04	Traces ^2^
Caftaric acid	Traces ^2^	8.81 ± 0.08	Traces ^2^
*t*-Coutaric acid	2.67 ± 0.09 ^a^	9.95 ± 0.06 ^b^	Traces ^2^
Caffeic acid	1.44 ± 0.01 ^a^	9.13 ± 0.03 ^b^	1.60 ± 0.01 ^c^
Epicatechin	0.66 ± 0.02 ^a^	0.44 ± 0.12 ^b^	0.76 ± 0.04 ^c^

^1^ Different letters in the same row indicate significant differences at 95% confidence according to the ANOVA and pairwise test. ^2^ Content below the LOQ. R: Regular winemaking process, with no freezing step. M: Winemaking process with the freezing of must (grape pulp and skins) after destemming and crushing. B: Winemaking process with the freezing of whole bunches of grapes. C: Winemaking with pre-fermentative cold maceration.

## Data Availability

Data is contained within the article.
